# Carotenoids and retinoids in the gonad of brood-stock pikeperch: accumulation during vitellogenesis and influence on egg quality in farmed pikeperch *Sander lucioperca*

**DOI:** 10.1590/1984-3143-AR2022-0103

**Published:** 2023-05-15

**Authors:** Sven Wuertz, Axel Orban, Fabian Johannes Schaefer, Julia Lynne Overton, Angela Krüger

**Affiliations:** 1 Leibniz Institute of Freshwater Ecology and Inland Fisheries, Berlin, Germany; 2 AquaPri Denmark A/S, Egtved, Denmark

**Keywords:** Sander, egg quality, vitellogenesis, retinol, hatching rate

## Abstract

Carotenoids are determinants of reproductive fitness and egg quality. Here we studied the accumulation of astaxanthin (AX), canthaxanthin (CA) zeaxanthin (ZX), lutein (LU), retinol (RX) and dehydroretinol (DR) during vitellogenesis comparing previtellogenic and vitellogenic pikeperch (*Sander lucioperca*) eggs (n = 5 each), as well as selected tissues (liver, fat and muscles) in first süawning females (1176-1450 g). Futhermore, we compared egg batches with high (88-99% hatching rate, n = 5) or low (40-67% hatching rate, n= 5) egg quality. Vitellogenic follicles revealed higher concentrations of DR, RX, ZX and LU compared to previtellogenic follicles. Neither CA nor AX was detectable. In parallel, DR and RX were mobilized in the liver. In adipose and muscle tissue, comparing previtellogenic and vitellogenic females, no significant differences in carotenoid/retinoid content were observed. In high quality egg batches, both DR and RX were increased. LU was lower in high quality than in low quality eggs. In a conclusion, the amount of retinoids seems suboptimal in low quality egg batches and increased DR and RX are desirable in pikeperch. Since hypervitaminosis of retinoids can be problematic though, supplementation of the food with carotenoids, which can serve as precursors for retinoids, has to be carried out carefully.

## Introduction

Pikeperch *Sander lucioperca* is an emerging candidate for European aquaculture, highly valued by the consumers for its delicious and firm meat (Wuertz et al., 2012). In their natural habitat, pikeperch reproduce at late spring at temperatures between 8-16 °C, dependent on the latitude (Lappalainen et al., 2003). In recirculation aquaculture, under tightly controlled photothermal conditions, reproduction can be achieved year-round after a wintering period (Müller-Belecke and Zienert, 2008; Hermelink et al., 2011; Hermelink et al., 2013). Reproductive management of pikeperch broodstock hence allows for a continuous, year round market supply of this temperate freshwater species. Still, low egg quality remains a major obstacle in pikeperch aquaculture, limiting the supply of larvae and juveniles for year-round production in recirculating aquaculture systems (Schaefer et al., 2016a; Schaefer et al., 2016b; Policar et al., 2019).

It is assumed that animals cannot carry out *de novo* synthesis of carotenoids (Matsuno et al., 1985; Johnson and An, 1991). Microalgae, terrestrial plants and crustaceans are the most common carotenoid sources (García-Chavarría and Lara-Flores, 2013). In aquaculture, some of these sources have been exploited to promote a desirable coloration, most prominent in salmon aquaculture (Bjerkeng, 2000), but increasing evidence suggests that carotenoids play an important role in eggs and may influence the quality of the eggs (García-Chavarría and Lara-Flores, 2013).

Carotenoids are hydrophobic substances that are only slowly absorbed in the gastro-intestinal tract (García-Chavarría and Lara-Flores, 2013). Absorption depends not only on the release from the food matrix but also on the subsequent solubilization by bile acids and incorporation into lipid micelles (Castenmiller and West, 1998). The capacity of micelles to incorporate carotenoids may be one factor that limits carotenoid uptake. For this reason, dietary lipids have been considered to be important cofactors for carotenoid bioavailability. The gastrointestinal epithelium and the liver are the most important organs for the catabolic transformation and interconversion of carotenoids. Perciformes fish cannot synthesize astaxanthin from other carotenoids (Matsuno et al., 1985). Therefore, astaxanthin originates from the diet. Still, perciformes can convert astaxanthin to zeaxanthin (Matsuno et al., 1985). Due to their hydrophobic character, carotenoids cannot circulate freely in the plasma but are absorbed to transport molecules such as high density lipoproteins or vitellogenin (Ando and Hatano, 1988). Reductive and oxidative metabolic transformations play an important role (Schiedt, 1998). The liver and the intestine are considered one of the most important organs for carotenoid metabolism (Hardy et al., 1990; Metusalach et al., 1996; Aas et al., 1999).

In fish, development in early life stages is dependent on essential nutrients incorporated during gametogenesis (Schaefer et al., 2016b; Schaefer et al., 2018). Here, provitamin A carotenoids and retinoids are actively transported to the ovaries and incorporated into the maturing oocytes, presumably during vitellogenesis (Ando and Hatano, 1988, 1991). After conversion to active forms such as retinol (vitamin A1) and dehydroretinol (vitamin A2), they play key roles in the developing embryo, including cell differentiation (Segner et al., 1989; Lampert et al., 2003), oxidative stress (Bjerkeng and Johnsen, 1995; Bell et al., 2000), reproduction and fertility (Torrissen and Christiansen, 1995), growth (Christiansen et al., 1995; Christiansen and Torrissen, 1996), immunity (Amar et al., 2004; Anbazahan et al., 2014) and vision (Lampert et al., 2003). Since the free alcohol forms of vitamin A can be toxic to cells, many tissues, most notably the liver, adipose tissue and kidneys, store vitamin A1 and A2 as esters of long chain fatty acids (e.g. retinyl palmitate, retinyl stearate, vitamin A2 analogues dehydroretinyl palmitate and dehydroretinyl stearate) (O'Byrne and Blaner, 2013). To serve as provitamin A, carotenoids must be cleaved to retinol or other forms of vitamin A (Castenmiller and West, 1998). There are approximately 600 different carotenoids, but only about 50 of these have provitamin A activity (Olson, 1989), among them lutein and zeaxanthin (Torrissen and Christiansen, 1995). In freshwater fish, in which more vitamin A2 is normally present, lutein and zeaxanthin seem to be dominantly present in the eggs.

Here, we studied the accumulation of retinoids and carotenoids in the gonads during oocyte maturation, comparing previtellogenic and vitellogenic females. Identifying the major carotenoids in pikeperch follicles during maturation will allow targeted nutritional supplementation in pikeperch in the future and support the planning of such interventions. Furthermore, we compared ovulated egg batches with high and low egg quality to seek an explanation of reduced egg batch quality in pikeperch broodstock. These observations on carotenoids in low quality compared to high quality eggs highlight the importance of a balanced supply with dietary carotenoids and identify candidates for nutritional supplementation.

## Material and methods

All experiments complied with the EU Directive 2010/63/EU and the national legislation for Animal Care in Science and were approved by the IGB animal welfare committee.

### Experiment 1: previtellogenic and vitellogenic females

Two years old female pikeperch (1176-1450 g) were obtained from a RAS-based pikeperch farm (Landesforschungsanstalt für Landwirtschaft und Fischerei Mecklenburg*-*Vorpommern, Hohen Wangelin) and kept in six 500 l tanks (8 fish per tank) arranged in a recirculation system. Maturation was induced by wintering (6 weeks at 12°C) as previously described (Hermelink et al., 2011; Hermelink et al., 2013; Hermelink et al., 2017). After acclimatization of 21 d at 21°C, the temperature was cooled down at 2 °C/d until adjusted to 12°C. Fish were kept under a 12L:12D photoperiod and fed on a commercial cod diet (DAN-EX 1750, Dana Feed/Biomar: 16% carbohydrates, 17% fat, and 50% protein) at a daily ratio of 0.5% of whole body mass. Oxygen (> 90%), temperature and pH (7.5-8) were determined daily (HQ40d multi, Hach Lange GmbH, Germany) and nitrogen species (NO_3_^-^-N < 20 mg/L, NO_2_^-^-N < 0.02 mg/L, TAN < 0.2 mg/L) every 3 days.

**Figure 1:**
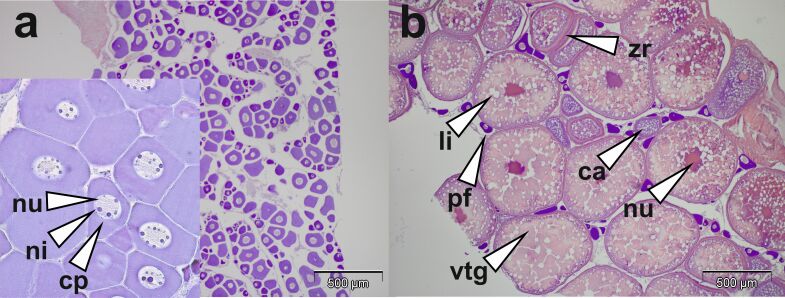
Previtellogenic and vitellogenic (mid vitellogenic) follicles of pikeperch *Sander luciperca*. Ca: cortical alveoli; cp: cytoplasma (basophilic in previtellogenic follicles); li: lipid droplets; ni: nucleoli; nu: nucleus; pf: previtellogenic follicle; vtg: vitellogenin globules; zr: zona radiate.

For sampling, fish were euthanized with 200 ppm MS222 and sampled (liver, gonad, adipose and muscle tissue) from a broodstock during thermal maturation a) before the wintering period at 23 °C and b) after six weeks of wintering at 12 °C, providing, respectively, five previtellogenic and five vitellogenic fish as confirmed by histological examination. In brief, gonad tissue was fixed in 4% phosphate-buffered formalin, embedded in paraplast, cut at 5 µm sections and stained with hematoxylin-eosin. The staging was performed according to Hermelink et al. (2011, 2013) with an Olympus RX50 microscope equipped with an Olympus XC50 digital camera (Figure 1).

### Experiment 2: high and low quality egg batches

Ovulated egg batches with high (88-99% hatching rate) and low quality (40-67% hatching rate) were sampled during routine reproduction at a commercial farm (AquaPri, Egtved, Denmark) as described previously (6, 7). In brief, the maturation of broodstock was induced by wintering below 14 °C for three months, followed by an increase to ~16 °C to complete final maturation without hormonal induction (6, 7). In female spawners, oogenesis was monitored by biopsies after anaesthetization (Kalmagin 20%; Centrovet, Chile). Ovulated females were anaesthetized, stripped and a subsample of the eggs was taken and stored in brown glass vials at -80 °C for the analysis of carotenoids and retinoids. The remaining eggs were fertilized with freshly stripped sperm according to methods described for pikeperch in Żarski et al. (2015). Survival and hatching of at least fifty eggs were assessed in triplicates (minimum 3* 50 eggs) to allow the identification of five females with low and high quality egg batches. Only these were considered here.

### Carotenoid and retinoid analysis

Liver, adipose, muscle and gonad tissue of previtellogenic and vitellogenic females (2.1) as well as ovulated eggs (2.2) were ground with a glass pestle. Appoximately 20 mg of the sample was extracted in 1 mL acetone after weighing it out with a Mettler AT261 Delta Range analytical balance. As an internal standard, 45 µL canthaxanthin (1 µg/ml methyl-tert-butylether (MTBE) / methanol solution, 1/1 V/V) was added, followed by homogenization in a TissueLyser (Qiagen Hilden, Germany) for 10 min at 8 Hz. After centrifugation (3 min, 20000 g, 4 °C), the supernatant was extracted three times in 1 mL acetone and frozen at -80 °C to separate lipids. After centrifugation (2 min, 2600 g, -9 °C), 1 mL was transferred to a new vial and concentrated with a vacuum concentrator (Eppendorf, Germany) in the V-HV mode for 20 min at 30 °C. For saponification of the remaining fat as well as hydrolysis of retinoid esters, 750 µL chloroform and 250 µL 20% methanolic KOH were added and samples were incubated for 1.5 h at 20 °C. After centrifugation (3 min, 20000 g, 4 °C), 750 µL was transferred to a new vial, 375 µL water was added, followed by intensive mixing and centrifugation (10 s, 20000 g, 4 °C). Washing with 375 µL was carried out three times and aqueous phases were pooled. Then, 500 µL chloroform was added, the organic phase was separated (10 s, 20000 g, 4 °C), the pooled organic fractions were concentrated with a vacuum concentrator and redissolved in 200 µL MTBE / methanol solution (1/1 V/V). In all steps nitrogen was used as carrier gas.

For the analysis of carotenoids and retinoids, astaxanthin (AX), canthaxanthin (CA) zeaxanthin (ZX), lutein (LU), retinol (RX) and 3,4 dehydroretinol (DR) were used as standards, dissolved at 10 µg/ml in MTBE /methanol solution (1/1 V/V) and stored in liquid nitrogen in the dark. Chromatographic separation was performed on an Agilent Zorbax Solvent Saver High Definition (RRHD) StableBond C18 column (3 x 100 mm, 1.8 µm, 1200 bar) using the 1290 Infinity UHPLC system from Agilent Technologies (Agilent, Santa Clara, CA, USA) equipped with a G7167B multi sampler. The mobile phase was composed of solution A) H_2_O (containing 0.1% formic acid) and solution B) acetonitrile (containing 0.1% formic acid) with a flow rate of 0.4 ml min^-1^, using the following gradient elution: 0-25 min 20% A, 80% B; 25-50 min from 80% to 95% B. The mass spectrometric analysis was carried out using the Agilent 6550 iFunnel QTOF with an Agilent Jet Stream electrospray ionization (ESI) source in positive mode. The sheath gas temperature was set to 400 °C with a flow of 11 l min^-1^, nebulizer at 45 psi and capillary voltage at 4000 V. Carotenoids and retinoids were identified and quantified with the MassHunter software targeting the most abundant molecule ions (Table 1). Standard dilution series were used to calculate the concentration of the respective target substance. The limit of detection and limit of quantification are given in Table 1.

**Table 1:** Assay specification of carotenoids and retinoids quantified. Most abundant molecule ions were identified with MassHunter software.

**Substance**	**Ion**	**m/z**	**LOD [ng/ml]**	**LOQ [ng/ml]**	**rel. error [%]**
Dehydroretinol	[M]^•+^	284.2135	0.12	0.38	2.16
Retinol	[M]^•+^	286.2291	0.38	1.27	2.89
Astaxanthin	[M+H]^+^	597.3938	1.01	3.34	2.10
Lutein	[M]^•+^	568.4275	1.60	5.29	1.68
Zeaxanthin	[M]^•+^	568.4275	1.68	5.56	1.80
Canthaxanthin	[M+H]^+^	565.4040	5.94	19.61	1.71

LOD: limit of detection; LOQ: limit of quantification.

### Data analysis

Data are presented as mean ± standard deviation (SD) of *n* samples. Data were tested for normality (Kolmogorov-Smirnov). For pair wise comparison, parametric Student’s t-test or non-parametric Mann-Whitney-U-test was performed. All statistical tests were carried out considering P < 0.05 using GraphPad Prism (GraphPad Software Inc., USA).

## Results

### Carotenoids and retinoids in previtellogenic and vitellogenic females

In previtellogenic females, follicles (80-100 µm) with basophilic cytoplasma and multiple nucleoli were observed (Figure1). Upon wintering, puberty was induced and vitellogenic follicles (500-800 µm) with a differentiated zona radiata interna and externa, peripheric cortical alveoli and vitellogenin containing vesicles as well as lipid globules were identified.

**Figure 2:**
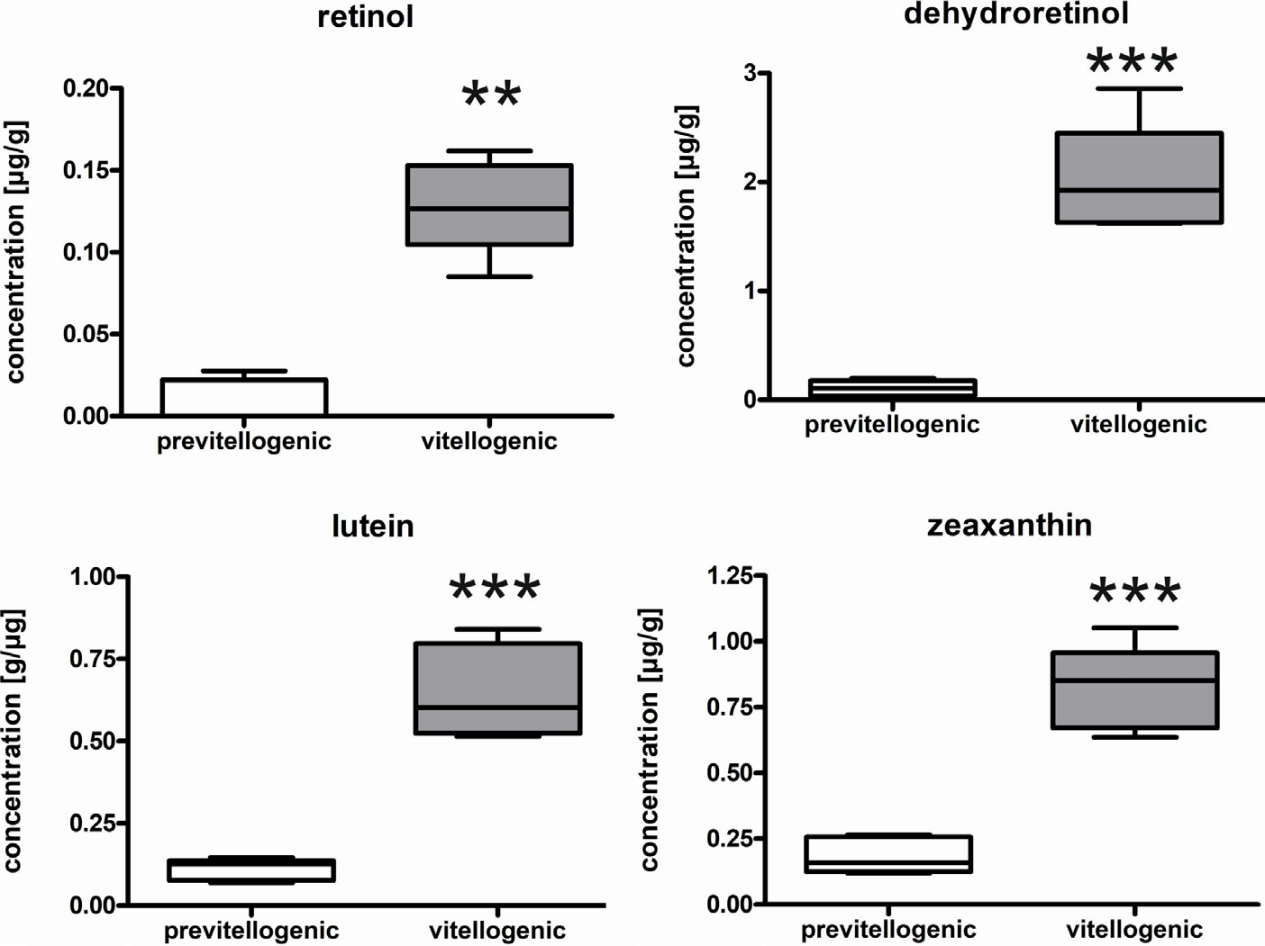
Carotenoids and retinoids, retinol, dehydroretinol, lutein and zeaxanthin lutein in the gonad of previtellogenic and vitellogenic ovaries. Significant differences are indicated by asterisk Student’s t-Test, ** p < 0.01, *** p< 0.001, n = 5).

**Figure 3:**
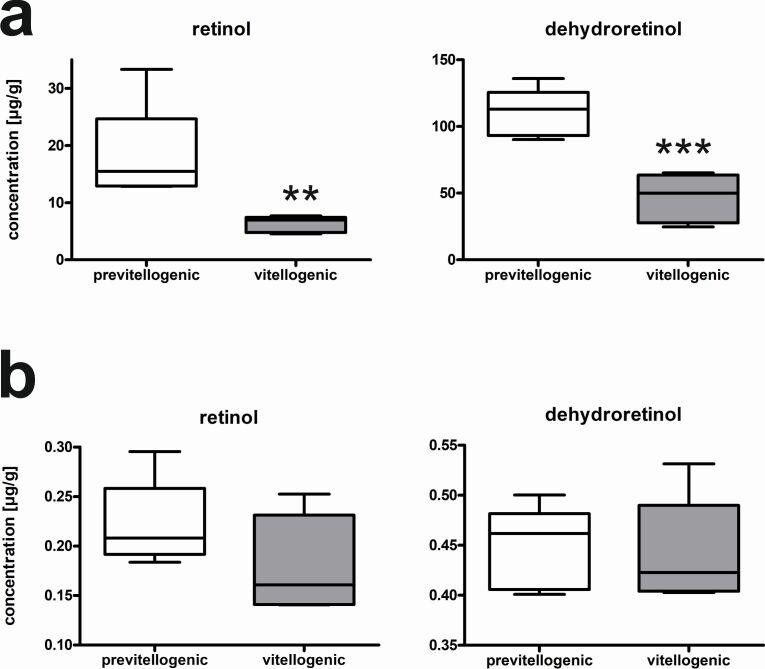
Retinoids, retinol and dehydroretinol in a) liver and b) fat tissue of previtellogenic and vitellogenic females. Significant differences are indicated by asterisk Student’s t-Test, ** p < 0.01, *** p< 0.001, n = 5). Lutein and zeaxanthin were below the detection limit.

RX increased from 0.01 ± 0.01 µg/g in previtellogenic to 0.13 ± 0.03 µg/g (p < 0.01) in vitellogenic follicles (Figure2). DR was the most abundant retinoid and increased from 0.11 ± 0.07 µg/g in previtellogenic to 2.02 ± 0.5 µg/g in vitellogenic follicles (p < 0.001). Similarly, LU and ZX were significantly higher in vitellogenic follicles (LU: 0.65 ± 0.14 µg/g, ZX: 0.82 ± 0.16 µg/g; p < 0.001) compared to previtellogenic follicles (LU: 0.11 ± 0.03 µg/g, ZX: 0.18 ± 0.07 µg/g; p<0.05). Thus, ZX was the most abundant carotenoid/retinoid (44%) in previtellogenic whereas in vitellogenic follicles DR prevailed. At the same time, the amount of RX and DR were significantly reduced in liver tissue from vitellogenic females (DR: 46.51 ± 18.20 µg/g, RX: 6.26 ± 1.40 µg/g) compared to previtellogenic females (DR: 110.06 ± 17.96 µg/g, RX: 18.12 ± 8.61 µg/g, p<0.05) (Figure3a). No significant differences in fat (Figure3b) and muscle tissue were observed between previtellogenic and vitellogenic females (previtellogenic adipose tissue: DR: 0.45 ± 0.04 µg/g, RX: 0.22 ± 0.04 µg/g; vitellogenic adipose tissue: DR: 0.44 ± 0.05 µg/g, RX: 0.18 ± 0.05 µg/g; previtellogenic muscles: DR: 0.008 ± 0.005 µg/g, RX: 0.002 ± 0.003 µg/g, LU: 0.001 ± 0.002 µg/g, ZX: 0.002 ± 0.003 µg/g; vitellogenic muscle: DR: 0.007 ± 004 µg/g, RX: 0.002 ± 0.004 µg/g, LU: 0.002 ± 0.002 µg/g, ZX: 0.003 ± 0.003 µg/g). In all tissues, neither AS nor CA were detected.

### Carotenoids and retinoids in high and low quality egg batches

**Figure 4:**
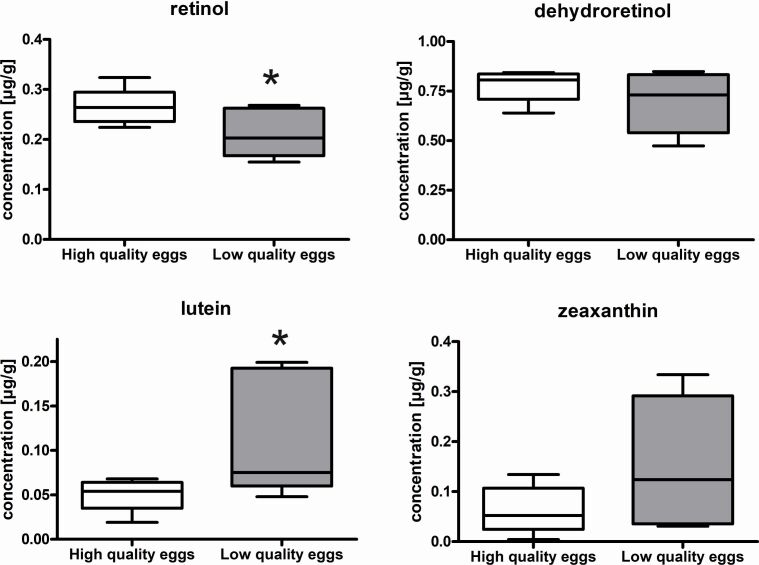
Carotenoids and retinoids, retinol, dehydroretinol, lutein and zeaxanthin in egg batches with high (88-99% hatching rate) or low (40-67% hatching rate) egg quality. Significant differences are indicated by asterisk (Student’s t-Test, * p < 0,05, n = 5).

Ovulated egg batches with high (88-99% hatching rate) quality revealed significantly elevated RX at 0.27 ± 0.04 µg/g compared to 0.21 ± 0.05 µg/g in low quality egg batches (40-67% hatching rate) (Figure4). Though not significant, DR was also higher (0.78 ± 0.08 µg/g) in high quality than in low quality eggs (0.7 ± 0.16 µg/g). Interestingly, LU was significantly lower in high quality than in low quality egg batches (0.05 ± 0.01 µg/g vs. 0.12 ± 0.07 µg/g). Also, though not significant, ZX was lower in high quality than in low quality egg batches.

## Discussion

In the past, most of the work on carotenoids focused on salmonids that accumulate high levels of AX in muscle as well as ovarian follicles (Torrissen et al., 1989; Ando and Hatano, 1991; Bjerkeng, 2000; Rajasingh et al., 2006; Tzanova et al., 2017). In salmonids, carotenoids improve fertility and reduce larval mortalities (Tzanova et al., 2017), but the role of carotenoids in pikeperch has not been studied. Indeed, information on percids mainly focus on marine species. Still, it has been reported that *Spirulina* supplemented diets with high carotenoid contents revealed higher egg production and better hatching rates in zebrafish *Danio rerio* (Güroy et al., 2012) and three-spot gourami *Trichopodus trichopterus* (Khanzadeh et al., 2016). Recently, lower vitamin A1 content was reported in the eggs of a captive pikeperch broodstock, strongly indicating that retinoid supply might often be insufficient in pikeperch farming (Mejri et al., 2017). Observations on the bioaccumulation of specific carotenoids during gonad maturation will therefore provide insights required for the timing of dietary interventions as well as providing arguments for the development of specific broodstock diets, required for the massive carotenoid accumulation into the gonad during maturation.

We established a LC-MS method to determine carotenoids and retinoids in different fish tissues. Integrating a saponification step, we were able to purify samples from extensive fat and include retinoid esters in our study. Making use of a relatively long LC run (50 min), we were able to differentiate the isomers LU and ZX in contrast to previous studies (Li et al., 2005). Li et al. (2005) used a protocol without saponification to quantify carotenoids in salmon. Using the same protocol, we encountered a reduced sensitivity due to massive matrix effects, emphasizing the need for a species-specific adaptation of protocols. In contrast to salmonids (Li et al., 2005; Rajasingh et al., 2006), AX and CA were not detected in pikeperch and CA was subsequently used as an internal standard.

Carotenoids and retinoids are stored at high concentrations in the body to buffer against nutritional insufficiency (Schiedt, 1998; García-Chavarría and Lara-Flores, 2013). Therefore, it can be hypothesized that the developing follicle is a site of carotenoid and retinoid bioaccumulation, providing the reserves for a developing embryo. Indeed, carotenoids are deposited in the eggs of several fish species (Torrissen et al., 1989). In pikeperch, elevated levels of DR, RX, ZX and LU were detected in vitellogenic ovaries suggesting that carotenoids as well as retinoids accumulate during the period of vitellogenesis, during or shortly after wintering. At the same time, RX and DR were over 100-fold increased in the liver, suggesting that the liver is the main storage site for carotenoids in pikeperch. During accumulation in the gonad, both retinoids are mobilized in the liver resulting in an overall decrease of retinoid reserves in the liver of vitellogenic females. This decrease in the main storage organ can be interpreted as an indication of a deficient dietary supply during maturation, advocating for a carotenoid supplemented diet in pikeperch broodstock. In vertebrates, retinol cannot be synthesized *de novo* and is stored mainly in the liver (Lubzens et al., 2003). Indeed the developing embryo crucially relies on the retinoids stored during vitellogenesis as reported in deprivation studies (Maden, 2001). The transport of rentinoids and carotenoids to the maturing eggs has therefore gained increasing attention. In general, carotenoids are transported by lipoproteins (LDL, HDL, VHDL) and albumin (Lubzens et al., 2003). Here, vitellogenin has been suggested to function as the main transporting protein. In contrast, retinoids are bound to the retinol-binding protein and -to a certain extend - to albumin for transport (Lubzens et al., 2003). Also, estradiol can induce the content of retinoids in the plasma (Palace et al., 2001a; Palace et al., 2001b), which may explain increased accumulation during estradiol-regulated vitellogenesis.

In Kutum *Rutilus frisii*, egg total carotenoid levels ranged between 6.6 and 7.9 µg/g (Alijanpour et al., 2015). In cultured rainbow trout, egg carotenoid concentrations of 2.8 µg/g were detected (Ando and Hatano, 1991). Here, LU and ZX were the most abundant carotenoids reported. In contrast, salmon mostly accumulate AX at concentrations between 6 and 8 µg/g (Ando and Hatano, 1991). In sander, we observed relatively low (< 2 µg/g) levels of carotenoids, similar to cultured rainbow trout *Oncorhynchus mykiss*. In contrast to salmonids, pikeperch has white flesh and carotenoid levels between 1-2 µg/g seem relatively high. Still, our study suggests that some increase may improve egg quality. In this context, it is important to keep in mind that supplementation may lead to hypervitaminosis. Indeed, chronic high vitamin A intake may lead to adverse effects in growth, fat deposition and bone formation and elevated vitamin A is a risk factor for bone deformities (Ørnsrud et al., 2002; Fontagné et al., 2006). Still, toxicity (hypervitaminosis) and deficiency often exhibit similar symptoms such as deformities (Ørnsrud et al., 2002), which are often observed in pikeperch hatcheries. In salmon, supplementation of retinol should not exceed 38 µg/g in the food (Ørnsrud et al., 2013). Consequently, supplementation has to be carried out carefully. Also, using carotenoids as supplements instead of retinol may help to reduce the risks of hypervitaminosis because carotenoids such as LU and ZX are incorporated into the pikeperch eggs and may be mobilized during development. In salmon fry, a concentration of 6 µg/g was considered safe and a slightly lower concentration may represent a safe approach for supplementation in sander (Ørnsrud et al., 2002).

Interestingly, neither ZX nor LU was detected in the liver. Indeed it is well established that carotenoids are mainly converted to retinol in the enterocytes of the gut (Castenmiller and West, 1998). Congruently, retinoids are the main storage form in maturing pikeperch. In fish as in mammals, it seems that the liver is the main vitamin A reserve, although other tissues, such as muscle in salmon, have been reported as storage sites for carotenoids/retinoids (Wold et al., 2004; Gesto et al., 2012). The low concentration of carotenoids/retinoids determined in the muscle during this study makes it unlikely that this tissue plays an important role in the storage of these substances in pikeperch. Furthermore, since pikeperch relies mainly on the stored retinoids in the liver, it should be considered relatively sensitive toward carotenoid deficient feed.

Until recently, it was thought that the sole important retinoid delivery pathway to tissues involved retinol bound to retinol-binding protein RBP4 (Lubzens et al., 2003; Levi et al., 2008). However, it was also reported that carotenoids and retinoids can be delivered via multiple delivery pathways, involving very high density lipoprotein (VHDL), high density lipoprotein (HDL), low density lipoprotein (LDL) as well as albumin (Ando and Hatano, 1991; Lubzens et al., 2003). Ando et al. (1986) reported that vitellogenin is the main protein associated with carotenoids in the VHDL, which suggests a close link between vitellogenesis and the bioaccumulation of carotenoids/retinoids. Congruently, we observed increased bioaccumulation of carotenoids and retinoids by the vitellogenic follicle. Interestingly, at the same time, neither ZX nor LU were detectable in the liver and fat tissue. This may indicate that carotenoids in the ovary originate from conversion processes either in the liver or the ovary itself, since ZX as well as LU were detected in vitellogenic follicles. Considering the presence of both carotenoids in the ovary, direct supplementation via the feed seems plausible. ZX and LU are among the most common carotenoids, found in plants, algae and microorganisms (García-Chavarría and Lara-Flores, 2013; Ram et al., 2020; Gupta et al., 2021; Sathyaruban et al., 2021). ZX could be supplemented with ingredients such as maize, paprika or Spirulina (Güroy et al., 2012), LU is found in pot marigold, spinach or nasturtium (Costa and Miranda, 2020).

In pikeperch, low gamete quality is the main obstacle to the development of the aquaculture industry and supply with stocking material is limited (Schaefer et al., 2016a; Schaefer et al., 2016b; Schaefer et al., 2018). In the present study, we compared concentrations of DR, RX, LU and ZX in egg batches of high and low quality. Here, a positive relationship was observed between the RX and hatching rates. Similarly, though not significant, DR was increased in high quality oocytes. Conversely, in low quality eggs, the carotenoids LU and ZX were increased. This indicates individual differences in the carotenoid/retinoid conversion probably due to intraspecific genetic differences important for breeding. Indeed, differences in carotenoid egg content have also been reported on the population level in other species (Castenmiller and West, 1998; Honeyfield et al., 2012; Alijanpour et al., 2015). Here, the increase in DR and RX in high quality eggs is in the range of the lower level of LU and ZX. One may therefore conclude that not the absolute concentration of DR and RX but rather a limited conversion of LU and ZX to DR and RX are linked to low quality of egg batches.

Carotenoids are both free radical scavengers and quenchers of singlet oxygen and have been shown to play a protective role against the action of free radicals (Choe and Min, 2009). Thus, carotenoids/retinoids can protect cell membranes and membrane integrity during early development. Still, AX, which is up to ten times stronger as an antioxidant than LU and ZX (Miki, 1991; Tzanova et al., 2017), was not detected here. Also, Schaefer et al. (2016b) did not observe oxidative stress in the low quality eggs ofpikeperch, assessing TAC and mitochondrial DNA fragmentation.

The functions of retinoids comprise important regulatory signaling during embryogenesis including cell division, growth and differentiation of tissues and, if limited, may therefore impact the survival of the developing embryo (Lubzens et al., 2003). Further investigations are needed, but increased levels of RX and DR in high quality eggs suggest that dietary carotenoids can improve the quality of egg batches in pikeperch. Also, a decrease in retinoid and carotenoid resources during vitellogenesis in the liver indicate a potential deficiency that requires specific broodstock nutrition. Indeed, the importance of carotenoids for the nutrition of maturing females has not received enough attention in the past. It is still unknown which carotenoid/retinoid species is most effectively bioaccumulated by the developing follicle and is thereby best suited for supplementation.
